# Combined Effect of *Bifidobacterium longum* Postbiotics and Dietary Herbs on Ameliorating Metabolic Disturbances in Hyperlipidemic Mice

**DOI:** 10.3390/foods15101679

**Published:** 2026-05-11

**Authors:** Yi Sun, Yihong Zeng, Ziyi Yue, Hang Yang, Yunhui Zhang, Haoxin Cui, Haiwei Liu, Hua Xiao, Jin Wang, Dancai Fan, Bowei Zhang, Huan Lv, Shuo Wang

**Affiliations:** 1 Tianjin Key Laboratory of Food Science and Health, School of Medicine, Nankai University, Tianjin 300071, China; 9920220161@nankai.edu.cn (Y.S.); yihongzeng@tju.edu.cn (Y.Z.); 2120251843@mail.nankai.edu.cn (Z.Y.); 2120231777@mail.nankai.edu.cn (H.Y.); zhangyunhui@mail.nankai.edu.cn (Y.Z.); cuihaoxin@mail.nankai.edu.cn (H.C.); haiweiliu927@163.com (H.L.); 2120231773@mail.nankai.edu.cn (H.X.); wangjin@nankai.edu.cn (J.W.); fandancai@163.com (D.F.); bwzhang@nankai.edu.cn (B.Z.); lvhuan@nankai.edu.cn (H.L.); 2 Research Institute of Public Health, Nankai University, Tianjin 300071, China

**Keywords:** postbiotics, dietary herbs, functional food, hyperlipidemia, bile acids, gut–brain axis

## Abstract

Hyperlipidemia-associated obesity is frequently accompanied by hepatic injury, bile acid dysregulation, gut microbial remodeling, and anxiety-like behavioral alterations. As emerging functional food ingredients, postbiotics and dietary herbs (DH) may provide practical dietary strategies for metabolic health management, but the most suitable postbiotic form and its compatibility with DH remain unclear. In this study, FB 3-14-derived postbiotics were first screened in vitro for cholesterol micellar binding. Inactivated bacterial cells (Postcell) exhibited the strongest cholesterol-binding capacity and were therefore selected for in vivo validation, alone or in combination with DH, in a high-fat, high-cholesterol (HFHC) mouse model. Consistently, Postcell showed superior efficacy in attenuating body weight gain, jejunal triglyceride accumulation, and hepatic dysfunction compared with other postbiotic forms. Importantly, Postcell_DH exerted broader metabolic benefits, including reductions in weight gain, food efficiency, bile acid dysregulation, and neuroinflammation. Multi-omics analysis further indicated that these effects may be mediated through remodeling of the gut microbiota and metabolome, particularly pathways involved in bile acid and tryptophan metabolism. Notably, *Clostridioides* and taurochenodeoxycholate-7-sulfate were negatively associated with total cholesterol (TC) and leptin, whereas *Clostridium_sensu_stricto_1* and 3-Hydroxyindolin-2-1-sulfate were negatively correlated with brain inflammatory level, lipid, and bile acid-related index. This study supports a practical postbiotic–herbal combination strategy relevant to functional food and dietary supplement development for hyperlipidemia-associated metabolic disturbances.

## 1. Introduction

Hyperlipidemia is a metabolic abnormality characterized by abnormally elevated circulating lipids or lipoproteins, including total cholesterol (TC), triglycerides (TG), and low-density lipoprotein cholesterol (LDL), and is generally considered to be a major risk factor for cardiovascular diseases [1]. The intestine is a critical site for dietary cholesterol absorption and bile acid recycling, thereby linking intestinal lipid handling with hepatic metabolism [2]. Dysregulation of bile acid synthesis or circulation can disrupt cholesterol homeostasis, intensify lipid accumulation, and aggravate metabolic dysfunction and liver injury [3]. Increasing evidence further indicates that a high-fat, high-cholesterol (HFHC) diet can also induce neuroinflammation, neuronal injury, and anxiety-like behavioral alterations through gut microbiota remodeling and metabolic disruption [4].

In recent years, postbiotics have attracted increasing attention as promising interventions for metabolic disorders and as potential functional food ingredients [5,6]. Compared with live probiotics, postbiotics offer several advantages, including improved safety, greater stability, longer shelf life, and lower costs for handling and storage [7]. Postbiotics are generally defined as preparations derived from probiotic microorganisms that contain non-viable cells, their structural components, and/or metabolites while retaining biological activity [5]. Generally, postbiotics comprise a complex mixture of bioactive substances, including extracellular polysaccharides (EPS), cell wall components, cell-free supernatants and/or metabolites while retaining biological activity [7]. Accumulating evidence indicates that postbiotics possess considerable lipid-lowering potential, as they promote cholesterol clearance in vitro and improve lipid metabolism, hepatic steatosis, and gut dysbiosis in vivo [8,9,10]. Additionally, postbiotics have been suggested to exert broad signaling effects across multiple organs and tissues, further supporting their potential in systemic metabolic regulation [11]. However, these postbiotic forms are not necessarily functionally equivalent [12]. Different postbiotic forms, such as whole inactivated cells, total postbiotic preparations, and cell-free supernatants, may differ substantially in their capacities to bind cholesterol, interact with bile salts, and regulate inflammatory responses or gut–brain communication. Therefore, the respective advantages and limitations of different postbiotic forms in host metabolic regulation remain to be systematically clarified.

Dietary herbs (DH) are increasingly recognized as functional food resources for chronic metabolic disorders because of their beneficial effects on obesity, oxidative stress, inflammation, and gut microbial homeostasis, along with their emerging capacity to modulators of the gut–brain axis. Among these, ginseng has been widely reported to exert anti-obesity effects, partly through bioactive components such as ginsenosides and polysaccharides that interact with the gut microbiota [13]. In addition, ginseng has demonstrated protective effects against brain injury and neuroinflammation, including alleviation of neuronal damage and inflammatory responses in neurodegenerative diseases [14]. Longan fruit is another representative dietary herb with multifunctional bioactivity. It has been reported to regulate lipid metabolism and improve metabolic disorders such as nonalcoholic fatty liver disease, while also influencing neurotransmitter-related processes involved in mood regulation and exhibiting anxiolytic and antioxidant properties [15,16].

These findings suggest that DH may complement postbiotics by providing benefits beyond conventional metabolic regulation, particularly in the context of liver–gut–brain interactions. Recently, increasing attention has been given to postbiotics generated through probiotic fermentation of DH, which have shown promising health benefits [17,18]. In contrast, studies investigating the direct combination of heat-inactivated bacterial cells and herbal components remain relatively limited. Notably, this combination strategy offers several practical advantages, including improved quality control, precise dose standardization, greater formulation flexibility, and preservation of the native bioactive components from both sources, thereby supporting its application as a functional food-based intervention [19].

In the present study, we aimed to identify the most effective lipid-lowering form among FB 3-14-derived postbiotics and to investigate whether combining it with DH could provide broader protective effects against hyperlipidemia-associated metabolic and neurological disturbances. Different postbiotic forms were compared using in vitro and in vivo screening, after which Postcell, which showed the strongest cholesterol-binding activity, was selected for combined intervention with DH. The effects of this combination were comprehensively evaluated in a hyperlipidemia mouse model by examining adiposity, lipid accumulation, liver function and histopathology, bile acid homeostasis, behavioral alterations, and brain inflammation. Moreover, 16S rRNA sequencing and untargeted metabolomics were employed to characterize the changes in gut microbiota and cecal metabolites. This study provides a theoretical and practical basis for the development of postbiotic–herbal combination strategies for hyperlipidemia and its associated neuroinflammatory and behavioral alterations.

## 2. Materials and Methods

### 2.1. Postbiotics and Dietary Herb Preparation

The probiotic strain *Bifidobacterium longum* subsp. *infantis* FB 3-14 (FB 3-14) used in this study was originally isolated from the feces of healthy breastfed infants and preserved at the General Microbiology Center of the China General Microbiological Culture Collection Center (CGMCC; No. 25762). The strain was cultured strictly anaerobically at 37 °C in an anaerobic workstation using BS broth/agar medium. After 24 h of incubation, the bacterial suspension was adjusted to 1 × 10^9^ CFU/mL and designated as LiveBL. The PostBL was prepared by heat treatment in a water bath at 80 °C for 30 min, with minor modifications from a previously described method [8]. Subsequently, the cell-free supernatant (PostCFS) and inactivated bacterial cells (Postcell) were separated by centrifugation at 8000× *g* for 15 min. The absence of viable bacteria in all postbiotic preparations was confirmed by plate culture.

The DH formula was prepared based on previous studies [14,15,16,20,21] with minor modifications, and consisted of ginseng (15 g), longan fruit (15 g), lotus leaf (15), *Poria cocos* (15 g), Chinese yam (10 g), coix seed (10 g), *Polygonatum odoratum* (10 g), malt (10 g), white hyacinth bean (10 g), dried tangerine peel (10 g), and hawthorn (5 g). These herbs were selected based on accumulating evidence demonstrating their involvement in lipid metabolism, bile acid homeostasis, and gut–brain axis regulation, thereby supporting their use as a functional dietary formulation. All herbal materials were purchased from Kangmei Pharmaceutical Co., Ltd. (Shenzhen, China). The herbs were decocted or extracted according to the previously described protocol [15,16]. Briefly, the mixed crude herbs were soaked in water (at a sixfold volume relative to the total crude-drug weight) for 30 min, decocted for 40–45 min, filtered, and concentrated on a rotary evaporator. The final decoction was adjusted to a concentration equivalent to 1 g of crude herbs per mL, which served as the dosing basis for this study. For the combined intervention, the DH preparation was physically mixed with Postcell at a 1:1 (*v*/*v*) ratio immediately before administration.

### 2.2. UHPLC-MS/MS-Based Metabolite Profiling of Dietary Herb Extracts

To improve the compositional transparency and reproducibility of the dietary herb (DH) intervention, the chemical profile of the DH extract was analyzed by ultra-high-performance liquid chromatography coupled with tandem mass spectrometry (UHPLC-MS/MS). Small-molecule compounds were identified based on accurate mass, retention time, MS/MS fragmentation patterns, and database matching. For sample preparation, 500 μL of dietary herb extract was mixed with 500 μL of methanol, vortexed for 10 min, and centrifuged at 12,000 rpm for 10 min at 4 °C. The supernatant was filtered through a 0.22 μm membrane before analysis. 2-Chlorophenylalanine was used as the internal standard.

Chromatographic separation was performed on a Vanquish UHPLC system using a Zorbax Eclipse C18 column (2.1 mm × 100 mm, 1.8 μm). The column temperature was 30 °C, the autosampler temperature was 4 °C, the injection volume was 2 μL, and the flow rate was 0.3 mL/min. The mobile phases consisted of 0.1% formic acid in water (A) and acetonitrile (B). The gradient was programmed as follows: 5% B, 0–2 min; 5–30% B, 2–6 min; 30% B, 6–7 min; 30–78% B, 7–12 min; 78% B, 12–14 min; 78–95% B, 14–17 min; 95% B, 17–20 min; 95–5% B, 20–21 min; and 5% B, 21–25 min.

Mass spectrometry was performed on a Q Exactive HF mass spectrometer with electrospray ionization in both positive and negative ion modes. The source parameters were as follows: heater temperature, 325 °C; sheath gas, 45 arb; auxiliary gas, 15 arb; sweep gas, 1 arb; spray voltage, 3.5 kV; capillary temperature, 330 °C; and S-lens RF level, 55%. Data were acquired in full-scan/dd-MS2 mode over an m/z range of 100–1500, with resolutions of 120,000 for MS1 and 60,000 for MS2. The TopN 5 method and HCD fragmentation were used for MS/MS acquisition.

Metabolites were semi-quantified based on peak areas and normalized to the internal standard. Relative concentrations were calculated as: internal standard concentration/internal standard peak area × metabolite peak area × dilution factor, and expressed as μg/mL.

### 2.3. In Vitro Determination of Cholesterol-Binding and Antioxidant Activity

To preliminarily compare the functional characteristics of FB 3-14-derived postbiotic forms and to further evaluate the rationale for combining Postcell with DH, we assessed the cholesterol micellar binding and antioxidant capacities of LiveBL, PostBL, PostCFS, Postcell, DH, and Postcell_DH in vitro.

The cholesterol micelle solution consisted of 10 mmol/L sodium taurocholate, 10 mM cholesterol, 5 mM oleic acid, and 132 mM NaCl dissolved in PBS [22]. The solution was sonicated for 1 h to aid dissolution and incubated at 37 °C for 24 h. Each sample was separately added to 5 mL of cholesterol micelle solution and incubated at 37 °C for 2 h. Following centrifugation, the cholesterol content in the supernatant was determined using a commercial assay kit. The cholesterol adsorption rate was calculated using the following formula: cholesterol micelle solubility inhibition rate (%) = (sample cholesterol content−control cholesterol content)/sample cholesterol content × 100%.

The DPPH free radical scavenging activity was assessed according to a previously reported method [23]. Briefly, 1 mL of the DPPH solution (0.2 mmol/L) was separately mixed with each sample. The mixtures were homogenized and incubated at 37 °C in the dark for 30 min. The samples were then centrifuged at 4000× *g* for 5 min, and the absorbance of the supernatant was measured at 517 nm.

### 2.4. Animals and Experimental Design

Specific-pathogen-free C57BL/6J mice (6-8 weeks, male) were obtained from Vital River Laboratory Animal Technology Co., Ltd. (Beijing, China) and housed under controlled conditions with a temperature of 20–25 °C and relative humidity of 50–60%. After a 1-week acclimatization period, the mice were randomly divided into 8 groups (*n* = 10 per group): Control, HFHC, LiveBL, PostBL, PostCFS, Postcell, DH (1 g/kg), and Postcell_DH (1 × 10^9^ CFU/mL inactivated cells + 1 g/kg DH). The Control group received a standard diet, whereas the HFHC and treatment groups were fed an HFHC diet supplemented with different FB 3-14-derived forms or DH, either alone or in combination. The standard diet (D12450J, 3.85 Kcal/g, 10% fat) and the HFHC (D12109C, 4.51 Kcal/g, 40% fat, 1.25% cholesterol, 0.5% sodium cholate) were purchased from Xietong Co., Ltd. (Nanjing, China). Mice had free access to food and water throughout the study. Body weight was recorded weekly, and food intake was monitored regularly. Food efficiency ratio = weight gain/food intake. After 12-week treatment, mice were anesthetized and sacrificed to collect serum, liver, epididymal fat, brain, cecal contents, and ileum. All animal experiments were approved by the Institutional Animal Care and Use Committee of Nankai University (animal ethics test approval number: 2025-SYDWLL-000454).

### 2.5. Behavioral Assessment

Behavioral performance was assessed using the open-field (OF) and the elevated maze (EPM) test. In the OF test, mice were placed individually in the center of a square arena (50 cm × 50 cm) enclosed by 45 cm-high walls and allowed to explore freely for 5 min. Exploratory activity in the center zone, including center time and center distance, was recorded to reflect anxiety-like behavior and exploratory capacity. The apparatus was cleaned with 75% ethanol to remove any smell cues before testing. In the EPM test, each mouse was placed on the central platform facing a closed arm and allowed to explore the maze for 5 min. The percentage of time spent in the open arms and the number of open-arm entries were recorded to assess anxiety-related behavior.

### 2.6. Tissue Biochemical Analysis and Histological Examination

At the end of the intervention, serum, liver, intestine, adipose tissue, cecal contents, and brain tissues were harvested. Serum TC, TG, LDL-C, serum alanine aminotransferase (ALT) and aspartate aminotransferase (AST), jejunal TG, and hepatic, ileal, and serum bile acid levels were quantified using commercial kits. Leptin, CYP7A1 activity, and brain inflammatory cytokines (IL-1β and TNF-α) were measured using ELISA kits according to the manufacturers’ protocols.

Adipose tissue and liver were collected immediately after sacrifice, fixed in 4% paraformaldehyde, and embedded in paraffin. Paraffin sections were stained with hematoxylin and eosin (H&E) according to standard procedures. Liver sections were also stained with Oil Red O to evaluate lipid droplet accumulation. For Nissl staining, brain sections were immersed in Nissl staining solution for 2–5 min, followed by rinsing with running water, drying at 65 °C, and mounting with neutral resin for microscopic analysis. For histological analyses, each data point shown in the figures represents one mouse (n = 6 per group). For each animal, three sections were analyzed, and five non-overlapping microscopic fields were evaluated per section. Field-level measurements were first averaged to obtain one value per section, and section-level values were then averaged to generate one final value per mouse for statistical analysis. Liver histopathology was semi-quantitatively scored on a 0–4 scale based on hepatocyte injury, inflammatory cell infiltration, and lobular architecture disruption, and the detailed scoring criteria are provided in Table S1. Histological evaluation was performed in a blinded manner, and quantitative image analysis was conducted using ImageJ 1.54 software.

### 2.7. Gut Microbiota Analysis in Mice Fecal Samples

Fecal gut microbiota analysis was performed as previously described [24]. Briefly, total microbial DNA was extracted from mouse fecal samples and subjected to quality assessment. The V3–V4 regions of the bacterial 16S rRNA genes were amplified by PCR using the primer pair 338F (5′-ACTCCTACGGGAGGCAGCA-3′) and 806R (5′-GGACTACHVGGGTWTCTAAT-3′). Sequencing libraries were constructed from the extracted DNA and sequenced using the Illumina NovaSeq platform (Illumina, San Diego, CA, USA). After sequencing, raw reads were processed through quality filtering, trimming, merging, denoising, and clustering. Taxonomic classification was then conducted to characterize the microbial composition and relative abundance of each sample. Alpha diversity, beta diversity, and PCoA were analyzed using QIIME 2, and Spearman correlation analysis was performed to assess the relationships between gut microbiota and metabolic parameters.

### 2.8. Untargeted Metabolomics Analysis in Mice Cecal Contents

UHPLC-MS/MS analysis was conducted at Novogene Co., Ltd. (Beijing, China) using a Vanquish UHPLC system (Thermo Fisher, Germany) coupled to an Orbitrap Q Exactive™ HF or Orbitrap Q Exactive™ HF-X mass spectrometer (Thermo Fisher, Germany). Samples were separated on a Hypersil Gold column (100 × 2.1 mm, 1.9 μm) with a 12 min linear gradient at a flow rate of 0.2 mL/min. For both positive and negative ion modes, the mobile phases consisted of eluent A (0.1% formic acid in water) and eluent B (methanol). The gradient program was as follows: 2% B at 1.5 min, 2–85% B from 1.5 to 3.0 min, 85–100% B from 3.0 to 10.0 min, 100% B from 10.0 to 10.1 min, and re-equilibration to 2% B until 12.0 min. The mass spectrometer was operated in both positive and negative electrospray ionization modes under the following conditions: spray voltage, 3.5 kV; capillary temperature, 320 °C; sheath gas flow rate, 35 psi; auxiliary gas flow rate, 10 L/min; S-lens RF level, 60; and auxiliary gas heater temperature, 350 °C. Raw UHPLC-MS/MS data were processed using XCMS for peak alignment, peak detection, and metabolite quantification. Peak intensities were first normalized to the total spectral intensity based on the first QC sample. Metabolite identification was then performed by matching the data against a high-quality secondary spectral database with a mass tolerance of 10 ppm and consideration of adduct ions, yielding qualitative and relative quantitative results. Statistical analyses were conducted using R (v.4.4.2), Python (version 2.7.6), and CentOS (release 6.6). For data that were not normally distributed, relative peak areas were normalized according to the following formula: sample raw quantitation value/(sum of metabolite quantitation values in the sample/sum of metabolite quantitation values in the QC1 sample). Compounds with coefficients of variation greater than 30% in QC samples were excluded from further analysis.

### 2.9. Statistical Analysis

All statistical analyses and data integration were performed in R (v4.4.2). Data are expressed as mean ± standard deviation (SD). Independent multi-group comparisons are described using one-way ANOVA with Dunnett’s multiple comparisons test. Gut microbial richness and evenness were assessed by α-diversity indices, while differences in community structure were visualized by PCoA. For metabolomics, identified compounds were annotated using the KEGG database. Differential metabolites were screened based on |log2FC| > 1.0, VIP > 2.0, and *p* < 0.01. Associations among gut microbiota, metabolites, and phenotypic traits were evaluated by two-sided Spearman’s correlation analysis with BH-adjusted *p*-values. A value of *p* < 0.05 was considered statistically significant.

## 3. Results

### 3.1. UHPLC-MS/MS Profiling of the DH Extract

To provide basic chemical characterization of the DH intervention, three independently prepared batches of the DH extract were analyzed by UHPLC-MS/MS in both negative- and positive-ion modes. The chromatographic profiles showed a complex but generally reproducible chemical pattern across batches, with similar overall peak distributions and major signal features in both ion modes (Figure S1A,B), supporting the batch-to-batch consistency of the DH preparation.

The top 50 putatively annotated compounds, ranked by peak area and relative concentration in the negative- and positive-ion modes, are summarized in Tables S2 and S3, respectively. To further assess preparation reproducibility, six representative constituents of the DH formula were selected from the top 50 compounds ranked by relative concentration; namely, hesperidin, narirutin, nuciferine, procyanidin B2, ginsenoside Rf, and hordenine. Their relative concentrations showed generally consistent patterns among the three independently prepared batches (Figure S1C,D), further supporting the compositional stability of the DH extract under the current preparation conditions.

### 3.2. In Vitro Function Screening of FB 3-14’s Postbiotics or Combination with Dietary Herbs

**Figure 1:**
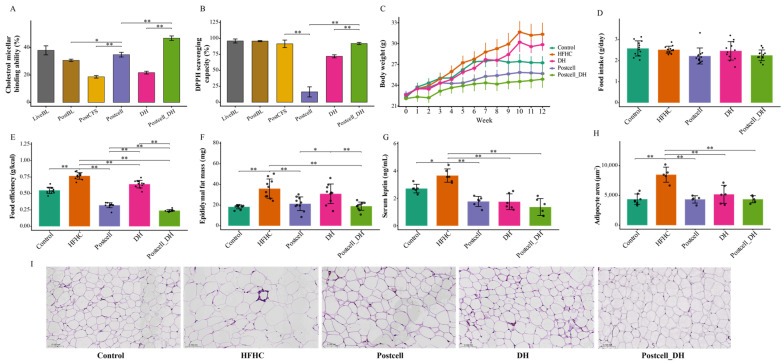
In vitro function screening of FB 3-14-derived postbiotics, alone or in combination with dietary herbs, and their anti-obesity effect in hyperlipidemia mice. (**A**) Cholesterol micellar binding ability, (**B**) DPPH radical scavenging capacity (%), (**C**) Body weight over the entire experimental period, (**D**) Food intake, (**E**) Food efficiency, (**F**) Epididymal fat mass, (**G**) Serum leptin levels, *n* = 10. (**H**) Quantification of adipocyte area, (**I**) Representative hematoxylin and eosin (H&E)-stained images of epididymis adipose tissue, *n* = 6. Data are presented as mean ± SD. * *p* < 0.05, ** *p* < 0.01.

To preliminarily compare the functional characteristics of FB 3-14-derived postbiotic forms and to further evaluate the rationale for combining Postcell with DH, we assessed cholesterol micellar binding ability and DPPH radical-scavenging capacity in vitro (Figure 1A,B).

Among the tested postbiotic preparations, Postcell showed relatively strong cholesterol micellar binding activity, whereas its antioxidant capacity was the lowest, as reflected by markedly reduced DPPH radical-scavenging activity. By contrast, DH alone showed limited cholesterol micellar binding ability but substantially higher antioxidant activity than Postcell. Notably, the combined Postcell_DH preparation exhibited improved cholesterol micellar binding and antioxidant performance compared with Postcell alone. These findings indicate that Postcell represents a more suitable lipid-oriented postbiotic form, whereas DH may complement its relatively weak antioxidant profile.

However, the in vitro screening was used only as a preliminary step to identify a lipid-oriented candidate, rather than as direct evidence explaining the complex in vivo outcomes. Therefore, the subsequent in vivo experiments were designed to validate Postcell as the preferred postbiotic candidate and to test whether functional complementation with DH could broaden the beneficial effects of the intervention under HFHC conditions.

### 3.3. Combined Effect of FB 3-14’s Postcell and Dietary Herbs on Fat Accumulation

Body weight changes over the 12-week experimental period are shown in Figure 1C. Compared with the Control group, mice in the HFHC group exhibited a significant increase in body weight after 7 weeks (*p* < 0.01). Moreover, the HFHC group showed the highest food efficiency, serum leptin level, epididymal fat mass, and adipocyte area (*p* < 0.05; Figure 1E–H), confirming the successful establishment of the diet-induced obesity. However, food intake did not differ significantly among groups (*p* > 0.05; Figure 1D), suggesting that the increased body weight and adiposity in the HFHC group were more likely related to altered energy utilization rather than greater food consumption.

No significant difference in body weight gain was observed between the LiveBL and PostBL groups, although the LiveBL group showed lower food efficiency (*p* < 0.01; Figure S2A,B). Among all FB 3-14-derived postbiotics, the Postcell group maintained the lowest body weight throughout the intervention period (Figure S2A,B). Notably, the Postcell_DH led to a pronounced suppression of weight gain after 1 week, compared with the Control, HFHC, and all postbiotic groups (Figure 1C). Consistently, the Postcell_DH group also showed the lowest food efficiency and reductions in serum leptin, epididymal fat mass, and adipocyte hypertrophy (*p* < 0.01; Figure 1E–H), highlighting a potentiated anti-obesity effect conferred by DH supplementation.

Collectively, among the different FB 3-14-derived postbiotic forms, Postcell exhibited potential effects on body weight control and food efficiency. Moreover, the combined treatment may provide a more comprehensive improvement in HFHC-induced adiposity under the tested condition.

### 3.4. Combined Effect of FB 3-14’s Postcell and Dietary Herbs on Lipid Metabolism

**Figure 2:**
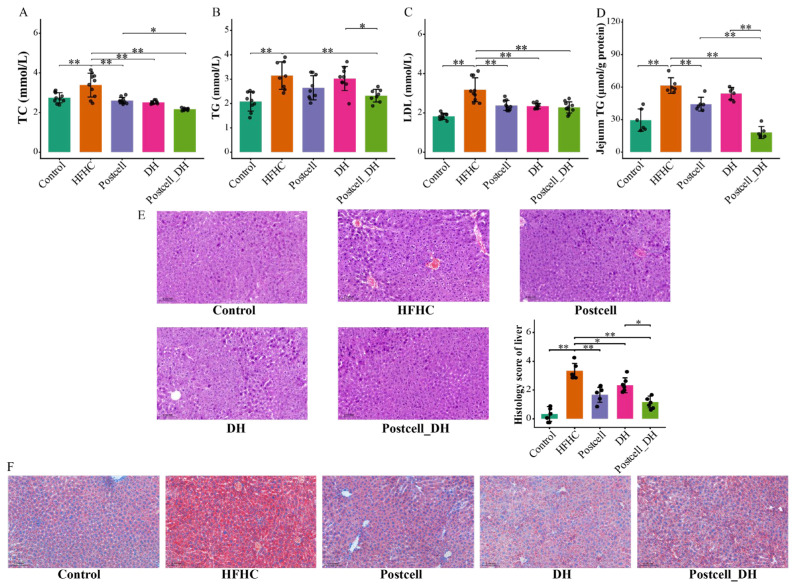
Effect of FB 3-14-derived postbiotics and dietary herbs on lipid metabolism. (**A**) Serum TC, (**B**) Serum TG, (**C**) Serum LDL, *n* = 10. (**D**) Jejunum TG, (**E**) Representative histologic images of hematoxylin and eosin (H&E) staining of liver sections and Histology score of the liver, (**F**) Representative Oil Red O-stained images of liver sections, *n* = 6. Data are presented as mean ± SD. * *p* < 0.05, ** *p* < 0.01.

To evaluate the effects of different interventions on lipid metabolism, serum lipid profiles and intestinal lipid accumulation were assessed. The HFHC diet significantly elevated serum TC, TG, LDL, and jejunum TG compared with the Control group (all *p* < 0.01; Figure 2A–D), confirming the successful establishment of the hyperlipidemia model. Among all postbiotics, Postcell showed a strong lipid-lowering effect, particularly in reducing jejunal TG accumulation, indicating an advantage in improving intestinal lipid deposition (*p* < 0.01; Figure S2H). Additionally, the Postcell_DH group demonstrated the most pronounced improvement with either treatment alone (Figure 2A,B,D). Specifically, jejunum TG content was most effectively reduced in the Postcell_DH group, suggesting that DH supplementation enhanced the lipid-lowering efficacy of Postcell, especially in limiting intestinal lipid accumulation and improving systemic lipid homeostasis.

Histopathological evaluation of liver sections by H&E staining further supported these biochemical findings. In the Control group, hepatic lobular architecture was intact, and hepatocytes were regularly arranged without obvious pathological alterations. In contrast, the HFHC group showed evident hepatic injury, characterized by disorganized hepatocyte arrangement, increased vacuolar degeneration, and inflammatory cell infiltration. Both Postcell and DH treatment alleviated these pathological changes, whereas the Postcell_DH group exhibited the most marked improvement, with a more preserved hepatic architecture, fewer vacuolated hepatocytes, and reduced inflammatory infiltration (Figure 2E). Consistently, liver oil Red O staining further showed that hepatic lipid droplet accumulation induced by the HFHC diet was visibly ameliorated, with the most pronounced improvement observed in the Postcell_DH group (Figure 2F).

Taken together, these results indicate that Postcell had a remarkable effect on intestinal and systemic lipid metabolism, and that its combination with DH produced broader benefits, resulting in a more comprehensive improvement in lipid metabolic disorders and liver pathological damage.

### 3.5. Combined Effect of FB 3-14’s Postcell and Dietary Herbs on Liver Function and Bile Acid Metabolism

**Figure 3:**
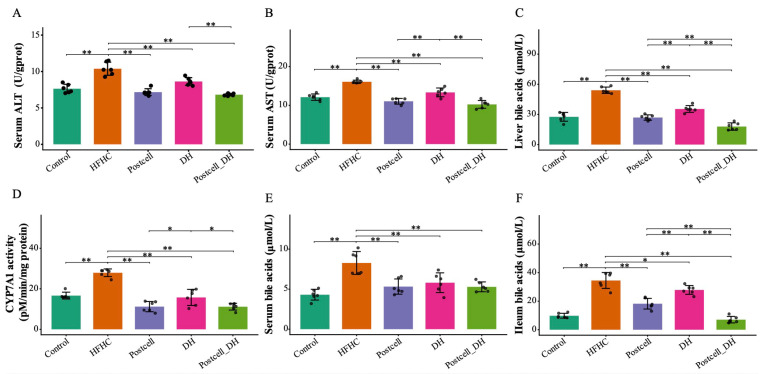
Effect of FB 3-14-derived postbiotics and dietary herbs on liver function and bile acid metabolism. (**A**) Serum ALT, (**B**) Serum AST, (**C**) Liver bile acids, (**D**) CYP7A1 activity, (**E**) Serum bile acids, (**F**) Ileum bile acids, *n* = 6. Data are presented as mean ± SD. * *p* < 0.05, ** *p* < 0.01.

Compared with the Control group, mice fed the HFHC diet showed significantly elevated serum ALT and AST levels, indicating liver injury under HFHC feeding (*p* < 0.05; Figure 3A,B). The HFHC diet also markedly increased total bile acid concentrations in the liver, ileum, and serum, and was accompanied by a significant elevation in hepatic CYP7A1 activity, suggesting expansion of the bile acid pool together with enhanced cholesterol-to-bile acid conversion (*p* < 0.01; Figure 3C–F).

Among all FB 3-14’s postbiotics, Postcell exerted the strongest hepatoprotective and bile acid-modulating effects, reducing serum AST levels, liver bile acid accumulation, and CYP7A1 activity (*p* < 0.05; Figure S2J–L). Notably, the combination of Postcell with DH further lowered hepatic and ileal bile acid levels compared with either treatment alone (*p* < 0.05; Figure 3C,F), indicating enhanced regulation of bile acid homeostasis.

These findings suggest that the efficacy of Postcell, especially when combined with DH, is closely linked to improved regulation of cholesterol catabolism and enterohepatic bile acid circulation, thereby contributing to protection against HFHC-induced hepatic metabolic injury.

### 3.6. Combined Effect of FB 3-14’s Postcell and Dietary Herbs on Brain Function

**Figure 4:**
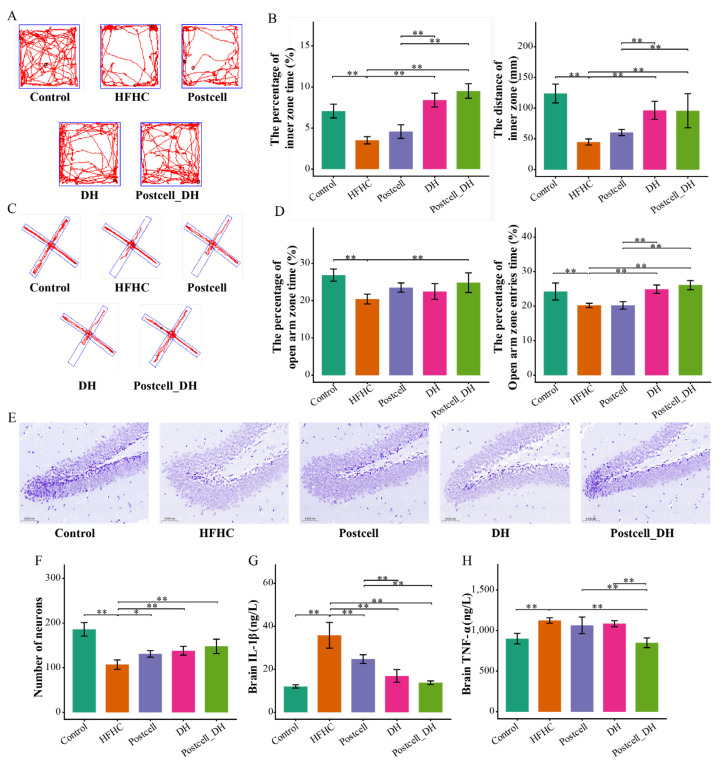
Effect of FB 3-14-derived postbiotics and dietary herbs on brain function and neuroinflammation. (**A**) Representative trajectories in the open-field (OF) test, (**B**) Time spent in the center area and total distance traveled in the OF test, (**C**) Representative trajectories in the EPM, (**D**) Time spent in open arms and the number of entries into open arms, (**E**) Nissl staining of the hippocampal region, (**F**) Number of neurons, (**G**) Brain IL-1β, (**H**) Brain TNF-α, *n* = 6. Data are presented as mean ± SD. * *p* < 0.05, ** *p* < 0.01.

After 12 weeks of HFHC feeding, mice exhibited severe impairment in brain-related behavioral, histological, and inflammatory levels. In the OF test, HFHC-fed mice showed significantly reduced exploration of the center area, as reflected by lower center time and center distance (*p* < 0.05; Figure 4A,B). In the EPM test, the HFHC group displayed reduced open-arm exploration (*p* < 0.05; Figure 4C,D). Histological examination revealed hippocampal neuronal injury, including lighter staining, fewer neurons, and disrupted cellular organization (Figure 4E,F). In parallel, brain inflammatory cytokines, particularly IL-1β and TNF-α, were elevated in the HFHC group, indicating that the HFHC diet induced substantial neuroinflammation (*p* < 0.01; Figure 4G,H).

Among the FB 3-14-derived postbiotics, all postbiotic treatments reduced brain IL-1β relative to HFHC (*p* < 0.05; Figure S2O), but none clearly improved TNF-α (*p* > 0.05; Figure S2P), indicating that FB 3-14-derived postbiotics alone exerted limited effects on HFHC-induced brain inflammation. DH alone significantly improved behavioral performance in both the OF and EPM tests, as well as brain IL-1β levels (*p* < 0.01; Figure 4).

Notably, the combination of Postcell with DH produced more pronounced improvements in brain-related outcomes than either treatment alone. In the Postcell_DH group, hippocampal histological abnormalities were markedly alleviated, with clearer cellular morphology, more orderly arrangement, and better preservation of neuronal structure (Figure 4E,F). Moreover, although Postcell or DH alone did not significantly reduce brain TNF-α levels, the combined intervention significantly lowered TNF-α, indicating a stronger suppressive effect on neuroinflammation (*p* < 0.01; Figure 4H).

These findings indicate that FB 3-14-derived postbiotics alone provided only partial protection, whereas the combination of Postcell and DH was associated with broader improvements in brain-related outcomes. This pattern suggests that DH may complement the relatively limited efficacy of Postcell on gut–brain-axis-related regulation and neuroinflammatory changes under the tested condition.

### 3.7. Combined Effect of FB 3-14’s Postcell and Dietary Herbs on Gut Microbiota

**Figure 5:**
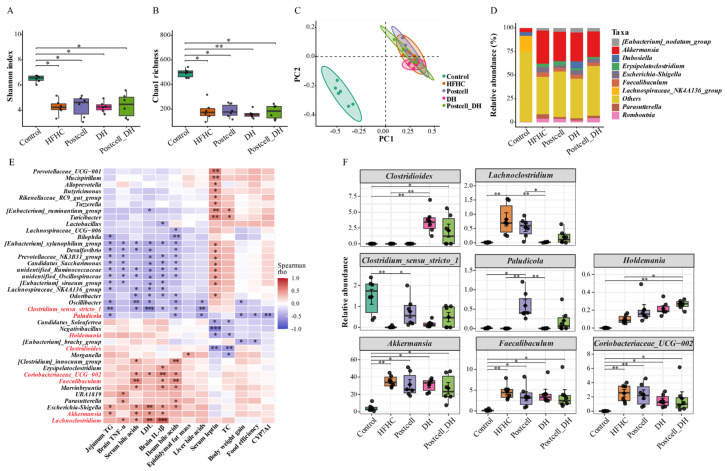
Effects of FB 3-14-derived postbiotics and dietary herbs on gut microbiota composition. (**A**) Shannon index, (**B**) Chao1 richness, (**C**) Beta diversity analysis based on Bray–Curtis distance (PCoA), (**D**) Relative abundance of dominant taxa at the genus level, (**E**) Correlation analysis between microbial taxa and metabolic parameters, (**F**) Differentially abundant microbial taxa among groups, *n* = 6. Data are presented as mean ± SD. * *p* < 0.05, ** *p* < 0.01, *** *p* < 0.001.

To determine whether the metabolic benefits of the interventions were associated with gut microbiota remodeling, 16S rRNA sequencing was performed. Compared with the Control group, the HFHC diet markedly reduced both the Shannon index and Chao1 richness, indicating a substantial loss of microbial diversity and richness. None of the interventions fully restored these alpha-diversity indices to Control levels (Figure 5A,B). Beta-diversity analysis showed clear separation between the Control and HFHC groups, confirming that HFHC feeding strongly altered community structure (Figure 5C). Among all intervention groups, only the DH group showed a significant difference from the HFHC group (PERMANOVA, *Padj* = 0.02), suggesting that DH exerted the most evident effect on microbiota structure.

At the genus level, the HFHC diet increased several taxa associated with a metabolic dysfunction, including *Akkermansia*, *Faecalibaculum*, and *Coriobacteriaceae_UCG-002* (Figure 5D,F). These genera were positively associated with bile acid circulation-related indices, brain inflammatory markers, and LDL levels (Figure 5E).

Compared with the HFHC group, DH treatment increased the relative abundance of *Clostridioides* and reduced *Lachnoclostridium* (*p* < 0.01; Figure 5F). Correlation analysis showed that *Clostridioides* was negatively associated with serum leptin and TC levels, whereas *Lachnoclostridium* was positively correlated with brain inflammatory markers, LDL, and serum bile acids (*p* < 0.05; Figure 5E). Moreover, Postcell treatment significantly increased the relative abundance of *Clostridium_sensu_stricto_1* and *Paludicola*, and this enrichment was specific to the Postcell intervention among the tested postbiotic forms (*p* < 0.05; Figure S2A). Correlation analysis further showed that both genera were negatively associated with several adverse phenotypes (*p* < 0.05; Figure 5E). The Postcell_DH group showed a similar tendency in these genera, although these changes did not reach statistical significance. Notably, the relative abundance of *Holdemania* was higher in the Postcell_DH group than in both the HFHC and Control groups (*p* < 0.05; Figure 5F). Correlation analysis indicated that *Holdemania* was significantly negatively associated with serum leptin and TC levels (*p* < 0.05; Figure 5E).

Collectively, these findings indicate that both Postcell and DH contributed to the partial restoration of microbial homeostasis, whereas the combined Postcell_DH intervention showed a broader corrective effect on HFHC-associated dysbiosis, shifting the gut microbiota toward a profile associated with improved lipid metabolism, bile acid homeostasis, and reduced neuroinflammation. These results suggest that gut microbiota remodeling may represent an important process associated with the broader metabolic benefits of the combined Postcell_DH treatment under the tested condition.

### 3.8. Combined Effect of FB 3-14’s Postcell and Dietary Herbs on Cecal Metabolites

To further elucidate the potential anti-obesity effects of Postcell, we first compared the HFHC and Postcell groups. A total of 277 cecal metabolites were significantly altered, including 148 downregulated and 129 upregulated metabolites in the Postcell group relative to the HFHC group (Figure S3B). Differential metabolites enriched in the Postcell group were associated with nucleotide metabolism and pyrimidine metabolism, represented by 4′-Thiothymidine, and also included Quercetol B and the lipid-related metabolites N-Lauroyl Methionine and N-Oleoyl Arginine (Figure S3C,D).

**Figure 6:**
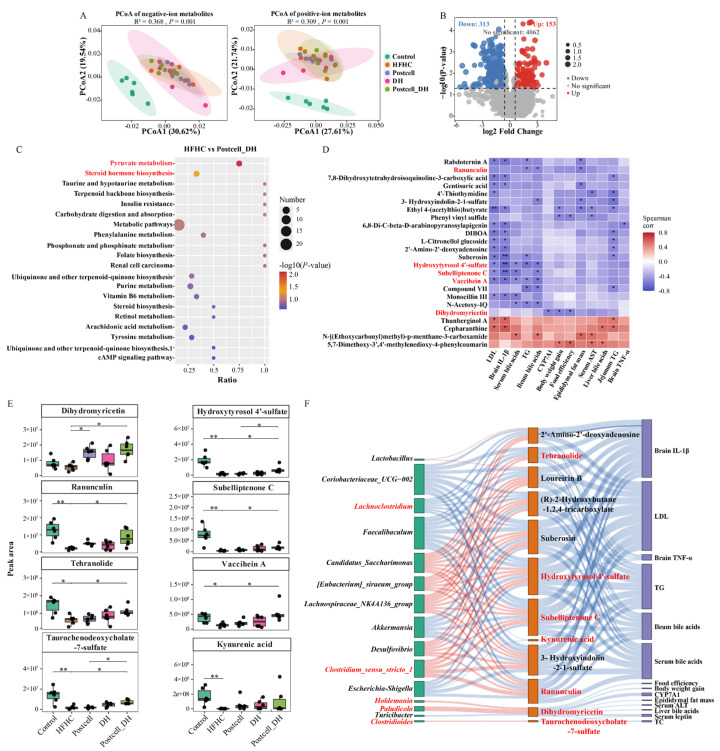
Effects of FB 3-14-derived postbiotics and dietary herbs on metabolomic profiles and multi-omics integration. (**A**) Principal coordinate analysis (PCoA) of metabolomic profiles. (**B**) Volcano plot of differential metabolites. (**C**) KEGG pathway enrichment analysis. (**D**) Heatmap of key differential metabolites. (**E**) The peak area of key differential metabolites (*n* = 6). (**F**) Correlation network of microbiota, metabolites, and metabolic phenotypes. Data are presented as mean ± SD. * *p* < 0.05, ** *p* < 0.01.

To further investigate the metabolic effects of the combined intervention, untargeted metabolomics was performed in the Control, HFHC, Postcell, DH, and Postcell_DH groups. Principal coordinates analysis based on Bray–Curtis distance demonstrated that the overall metabolomic profiles differed significantly among groups in both negative- and positive-ion modes (negative mode: R^2^ = 0.368, *p* = 0.001; positive mode: R^2^ = 0.309, *p* = 0.001). Pairwise PERMANOVA further showed that only the Postcell_DH group differed significantly from the HFHC group (R^2^ = 0.186, *Padj* = 0.045), indicating that the combined treatment induced the most pronounced shift in the metabolic profile (Figure 6A). Compared with the HFHC group, the Postcell_DH group showed 466 differential metabolites, including 313 downregulated and 153 upregulated features (Figure 6B). KEGG enrichment analysis highlighted pyruvate metabolism and steroid hormone biosynthesis among the significantly enriched pathways (Figure 6C).

Correlation analysis further identified metabolites associated with at least three hyperlipidemia-related phenotypes. Compared with the HFHC group, several metabolites were enriched in the Postcell_DH group, including hydroxytyrosol 4′-sulfate, ranunculin, subelliptenone C, tehranolide, vaccihein A, taurochenodeoxycholate-7-sulfate, and kynurenic acid. These metabolites also showed a relationship with adiposity, dyslipidemia, bile acid-related indices, and neuroinflammatory markers (*p* < 0.05; Figure 6D,E). Additionally, dihydromyricetin was increased in both the Postcell and Postcell_DH groups compared with the Control and HFHC groups (*p* < 0.05; Figure 6E).

Spearman correlation analysis was next performed to summarize the key correlations among microbial taxa, metabolites, and metabolic index (Figure 6F). *Clostridioides* and taurochenodeoxycholate-7-sulfate, both enriched in the Postcell_DH group, were positively correlated with each other and negatively associated with serum TC and leptin levels. Kynurenic acid was negatively correlated with *Escherichia-Shigella* and *Coriobacteriaceae_UCG-002*, as well as with LDL-related traits. In addition, 3-Hydroxyindolin-2-1-sulfate was positively associated with *Clostridium_sensu_stricto_1*, a taxon enriched in the Postcell group that was negatively correlated with brain inflammatory indices, lipid parameters, and bile acid-related traits. *Holdemania*, enriched in the Postcell_DH group, was positively associated with dihydromyricetin as well as CYP7A1, serum leptin, and TC. Together, these findings suggest that the beneficial metabolic effects of Postcell_DH may be mediated through coordinated remodeling of the gut microbiota–metabolite–phenotype interactions.

## 4. Discussion

Compared with live probiotics, postbiotics offer practical advantages, including improved safety, stability, shelf-life, and batch-to-batch consistency, supporting their use as functional food or dietary supplement ingredients for metabolic health management [7]. In our previous study, *Bifidobacterium longum* subsp. *infantis* FB 3-14 showed anti-obesity and lipid-lowering effects in high-fat-fed mice [24]. However, whether its postbiotic derivatives exert comparable benefits, and which postbiotic form is most suitable for intervening in hyperlipidemia-associated obesity, especially under conditions involving bile acid dysregulation, neuroinflammation, and anxiety-like behavior, remained unclear. In the present study, LiveBL and PostBL did not differ significantly in their effects on body weight gain, lipid metabolism, or brain inflammatory markers, suggesting that heat inactivation did not substantially impair its major metabolic benefits. Notably, however, PostBL produced a greater reduction in hepatic bile acid levels than LiveBL, indicating that postbiotic preparations may retain specific metabolic functions of the live strain, particularly in regulating bile acid homeostasis.

Different postbiotic forms are not necessarily functionally equivalent. In the present study, FB 3-14-derived postbiotics possess distinct functional properties in vitro and in vivo. Interestingly, inactivated bacterial cells (Postcell) exhibited the strongest cholesterol micellar binding activity in vitro and showed the clearest improvement in lipid-related phenotypes in vivo, including attenuation of body weight gain, reduction in jejunal TG accumulation, improvement of serum lipid profiles, and alleviation of hepatic injury and bile acid disturbance. Previous studies have shown that non-viable lactic acid bacteria (LAB) can remove cholesterol through surface binding, membrane incorporation, and bile-related interactions [25,26], while EPS produced by LAB also exhibits cholesterol-lowering effects in vitro [22,27]. Consistent with these findings, the lipid-related benefits of Postcell may be partly attributed to retained bacterial structural components after inactivation, which could interact with cholesterol micelles or bile salts in the intestinal lumen and thereby reduce intestinal lipid absorption, particularly in the jejunum. Moreover, Postcell treatment markedly reshaped the gut microbial composition, characterized by a significant enrichment of *Clostridium_sensu_stricto_1* and *Paludicola*. Previous studies have indicated that *Clostridium_sensu_stricto_1* is involved in bile acid metabolism [28], while *Paludicola* has been negatively associated with body weight and positively correlated with energy expenditure and insulin sensitivity [21]. In the present study, correlation analysis showed that these genera were negatively associated with jejunal TG levels and intestinal or hepatic bile acid concentrations, suggesting a potential link between Postcell-associated microbial changes and lipid or bile acid-related phenotypes. At the metabolomic level, Postcell intervention was accompanied by changes in cecal metabolites related to nucleotide and pyrimidine metabolism pathways, including 4′-thiothymidine. Several bioactive compounds, including Quercetol B and lipid-related metabolites such as N-lauroyl methionine and N-oleoyl arginine, were also enriched in the Postcell group, which may contribute to improved lipid handling and energy metabolism. Together, these findings suggest that Postcell improved body-weight control, jejunal TG accumulation, and liver-related outcomes. The associated microbiota and metabolite changes may help to explain these effects, but causal relationships require further validation.

However, the effect of Postcell alone on the gut–brain axis appeared relatively limited, possibly reflecting a narrower profile of soluble metabolites or signaling molecules compared with cell-free supernatant [29]. By comparison, DH alone showed pronounced neuroprotective and anti-inflammatory effects in the present study, which is consistent with its reported antioxidant, anti-inflammatory, and neuroregulatory activities [14,15]. Specifically, DH significantly improved performance in the OF and EPM test, suggesting enhanced exploratory behavior and reduced anxiety-like behavior in rodents. In addition, DH treatment was associated with better hippocampal neuronal preservation and reduced brain IL-1β levels, further supporting its protective effects against HFHC-induced neuroinflammation and neuronal injury. In this study, six abundant constituents were selected as representative markers for cross-batch comparison because they have been reported as characteristic or bioactive compounds of herbs included in the DH formula and are associated with metabolic, anti-inflammatory, or gut–brain-axis-related functions. These constituents included ginsenoside Rf (20.85 ± 0.51 μg/mL), nuciferine (276.51 ± 4.37 μg/mL), hesperidin (453.54 ± 4.88 μg/mL), narirutin (228.89 ± 2.01 μg/mL), procyanidins B2 (35.55 ± 0.53 μg/mL) and hordenine (3.88 ± 0.08 μg/mL) (Figure S1C and Table S2). Specifically, ginsenoside Rf has been linked to neuroprotective, anti-inflammatory, and metabolic regulatory effects [30], while nuciferine, a lotus leaf extract, has been reported to alleviate inflammatory and neuronal apoptosis [31,32]. Hesperidin and narirutin, two key bioactive compounds from tangerine peel, have been associated with improved lipid metabolism, anxiety- or depression-like behaviors, and AMPK-mediated energy metabolism [33,34]. Additionally, procyanidins B2, abundant in hawthorn [35], has been reported to attenuate aging-related behavioral changes and neuroinflammation by binding to TLR4 [36], whereas hordenine, predominantly found in malt, can protect dopaminergic neurons by reducing pro-inflammatory factors such as IL-6 and TNF-α [37]. Taken together, these findings indicate that DH may compensate for the relatively limited gut–brain regulatory activity of Postcell, broadening the functional spectrum of the combined intervention.

In the present study, the Postcell_DH combination exhibited more pronounced protective effects than either component alone, especially in cholesterol-lowering and antioxidant ability in vitro, as well as neuroinflammation, bile acid homeostasis, and metabolic regulation in vivo. This broader efficacy may be related to functional complementation between the two components. Specifically, Postcell may act at the intestinal level by modulating cholesterol handling and bile acid balance, whereas DH may have broader systemic effects, including antioxidant activity, neuroinflammatory regulation, and gut–brain axis protection. These findings highlight a feasible functional food-based strategy for integrating postbiotics and DH to achieve broader metabolic and brain-related benefits. Nevertheless, the specific mechanisms underlying the combined effects of Postcell and DH require further investigation through dose–response analysis and ratio optimization.

The small intestine is a key site for dietary cholesterol absorption and bile acid reabsorption, thereby linking intestinal lipid handling with hepatic metabolism through the enterohepatic cycle [38,39]. As cholesterol-derived metabolites, bile acids play a central role in maintaining lipid homeostasis. However, chronic HFHC feeding disrupts this balance by enhancing CYP7A1 activity, elevating bile acid synthesis and accumulation, which contributes to dyslipidemia and liver injury [2,3]. The HFHC diet used in this study contained 0.5% sodium cholate in addition to high fat and cholesterol. Because sodium cholate is itself a bile salt, it may directly perturb bile acid pool composition, enterohepatic circulation, hepatic injury, and gut microbiota structure. Thus, the bile acid dysregulation observed in the HFHC group should be interpreted as the result of a combined high-fat, high-cholesterol, and sodium-cholate dietary challenge. Consistently, in the present study, 12 weeks of HFHC feeding markedly elevated total bile acid levels in the serum, liver, and ileum, accompanied by increased CYP7A1 activity, indicating exaggerated cholesterol-to-bile-acid conversion and disrupted enterohepatic homeostasis. Notably, the Postcell_DH intervention partially alleviated these alterations, with significant reductions in hepatic and ileal bile acid levels and broader effects than either Postcell or DH alone under the tested condition. Given that CYP7A1 is the rate-limiting enzyme in bile acid synthesis, its downregulation, particularly in the Postcell_DH group, suggests that the combined treatment alleviated upstream cholesterol burden and suppressed excessive bile acid production.

In addition, both *Clostridioides* and taurochenodeoxycholate-7-sulfate were significantly enriched in the Postcell_DH group and showed a positive correlation with each other. Previous studies have demonstrated that *Clostridioides* may participate in bile acid metabolism through bile salt hydrolase-related activity, and that its growth and function can be influenced by the composition of the bile acid pool [40]. Taurochenodeoxycholate-7-sulfate, a sulfated bile acid derivative, has been implicated in bile acid detoxification and homeostasis. Consistent with our findings, Zhang et al. [41] reported that bile acid biosynthesis is a key pathway disrupted by the HFHC feeding. In particular, several primary bile acids, including taurocholic acid, taurochenodeoxycholic acid, glycocholic acid, and taurochenodeoxycholic acid, are markedly elevated. In the present study, the co-enrichment of *Clostridioides* with taurochenodeoxycholate-7-sulfate, together with their negative associations with serum TC and leptin, suggests a potential link between Postcell_DH-associated microbial changes, bile acid-related metabolites, and improved metabolic phenotypes. However, these relationships are based on correlation analysis and should be interpreted as associative rather than causal. Further studies are needed to determine whether *Clostridioides* directly contributes to bile acid transformation or metabolic improvement.

Increasing evidence suggests that chronic HFHC feeding may be accompanied not only by metabolic disorders, but also by anxiety-like behavior and neuroinflammation [4,42]. In the present study, the Postcell_DH intervention significantly improved anxiety-like behavior, neuronal preservation, and neuroinflammation. Notably, Postcell_DH markedly reduced brain TNF-α levels, whereas Postcell or DH alone showed no significant effect, indicating a stronger neuroprotective efficacy of the combined treatment. Multi-omics analysis further supported the involvement of tryptophan-related microbial metabolites in this process. Kynurenic acid (KYNA), an important metabolite of the tryptophan pathway, has attracted attention because of its immunomodulatory and neuroprotective properties. KYNA can interact with GPR35 expressed on macrophages and other immune cells, and is linked to the regulation of cytokine production and inflammatory responses [43]. In our study, KYNA was negatively correlated with *Escherichia-Shigella* and *Coriobacteriaceae_UCG-002*, as well as with LDL-related traits, suggesting a potential relationship between tryptophan-related metabolic changes, neuroinflammatory status, and lipid metabolism. Consistent with our results, *Coriobacteriaceae_UCG-002* has been proven to be associated with cognitive performance [44] and to participate in bile acid biotransformation, including 6β-epimerization and 7α-dehydroxylation [45]. In addition, 3-hydroxyindolin-2-one sulfate, another tryptophan-related metabolite, was positively associated with *Clostridium_sensu_stricto_1*, while this genus was negatively correlated with brain inflammatory index, lipid parameters, and bile acid-related traits. These associations suggest a possible link among gut microbiota, tryptophan-related metabolites, and host metabolic or neuroinflammatory phenotypes. However, these multi-omics findings are correlative, and further studies are required to determine whether these microbial taxa or metabolites directly contribute to the observed benefits of the combined treatment.

Our study highlights that different FB 3-14-derived postbiotic forms possess distinct functional properties and that the inactivated cell fraction represents a preferred candidate for lipid-targeted intervention. Moreover, it provides evidence that a physically combined postbiotic–herbal strategy achieves broader efficacy than either component alone, encompassing improvements in obesity, dyslipidemia, hepatic dysfunction, bile acid imbalance, gut microbial dysbiosis, neuroinflammatory, and anxiety-like behavioral alterations in hyperlipidemic mice.

However, some limitations need to be addressed in future research. First, the associations identified among gut microbiota, metabolites, and host phenotypes were primarily based on correlation analyses. The underlying causal relationships require further study. Second, because only one dose level and one mixing ratio were tested in the present study, and no formal interaction analysis was performed, the current data do not establish pharmacological or statistical synergy. Therefore, the combined intervention should be interpreted more cautiously as a functionally complementary strategy under the tested condition. Future studies incorporating multiple doses and formulation ratios are needed to better define dose–response relationships and clarify the specific contributions of Postcell and DH.

## 5. Conclusions

In this study, Postcell exhibited the strongest cholesterol-binding capacity in vitro and efficacy in attenuating body weight gain, jejunal TG accumulation, and hepatic dysfunction compared with other postbiotic forms. Moreover, Postcell_DH exerted broader metabolic benefits, including reductions in weight gain, food efficiency, bile acid dysregulation, anxiety-like behavior, and neuroinflammation. Multi-omics analyses suggested that the benefits were associated with alterations in the gut microbiota and metabolome, particularly pathways related to bile acids and tryptophan metabolism. Notably, *Clostridioides* and taurochenodeoxycholate-7-sulfate were negatively associated with TC and leptin, whereas *Clostridium_sensu_stricto_1* and 3-Hydroxyindolin-2-1-sulfate were negatively correlated with brain inflammatory level, lipid, and bile acid-related index. These findings support a practical strategy combining lipid-oriented postbiotic form with herbal functional complementation to ameliorate hyperlipidemia-associated metabolic, neuroinflammatory, and anxiety-like behavioral alterations.

## Data Availability

The original contributions presented in this study are included in the article/Supplementary Material. Further inquiries can be directed to the corresponding author.

## Supplementary Materials

The following supporting information can be downloaded at: https://www.mdpi.com/article/10.3390/foods15101679/s1, Figure S1: UHPLC-MS/MS-based chemical profiling and batch consistency of the dietary herb extract. Figure S2: Effect of different FB 3-14-derived postbiotics in mice fed a high-fat and high-cholesterol diet; Figure S3: Effect of different FB 3-14-derived postbiotics on microbiome and metabolomic profiles; Table S1: Histological Score of Liver Tissue; Table S2: Top 50 annotated features detected in the DH extract across three independently prepared batches (negative-ion); Table S3: Top 50 annotated features detected in the DH extract across three independently prepared batches (positive-ion).

## Abbreviations

The following abbreviations are used in this manuscript:

DH	Dietary herbs
HFHC	High-fat, high-cholesterol
TC	Total cholesterol
TG	Total triglycerides
LDL	Low-density lipoprotein cholesterol
EPS	Extracellular polysaccharides
OF	Open-field
EPM	Elevated maze
ALT	Alanine aminotransferase
AST	Aspartate aminotransferase
LAB	Lactic acid bacteria
KYNA	Kynurenic acid
