# Supplementary material for: Combined Effect of Bifidobacterium longum Postbiotics and Dietary Herbs on Ameliorating Metabolic Disturbances in Hyperlipidemic Mice
Source: Foods. 2026 May 11;15(10):1679. doi: 10.3390/foods15101679 (PMC13206221; doi:10.3390/foods15101679)
Supplement: Supplementary file 1 [file foods-15-01679-s001.zip › foods-4257106-supplementary.pdf]

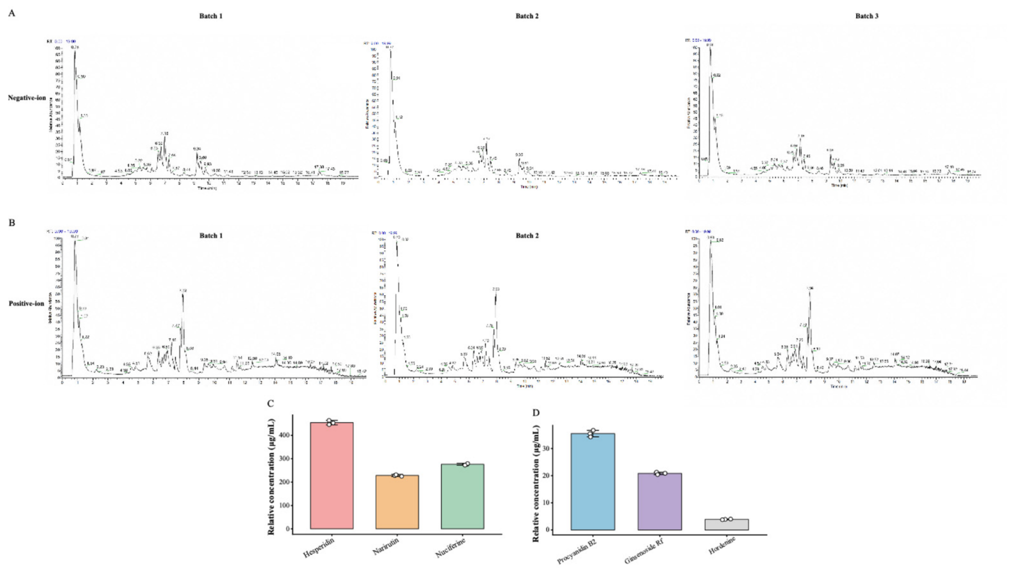

Figure S1. UHPLC-MS/MS-based chemical profiling and batch consistency of the dietary herb extract. **(A)** Negative-ion chromatograms of three independently prepared DH batches. **(B)** Positive-ion chromatograms of three independently prepared DH batches. **(C)** Relative concentrations of hesperidin, narirutin, and nuciferine across three DH batches. **(D)** Relative concentrations of procyanidin B2, ginsenoside Rf, and hordenine across three DH batches. Data are presented as mean ± SD.

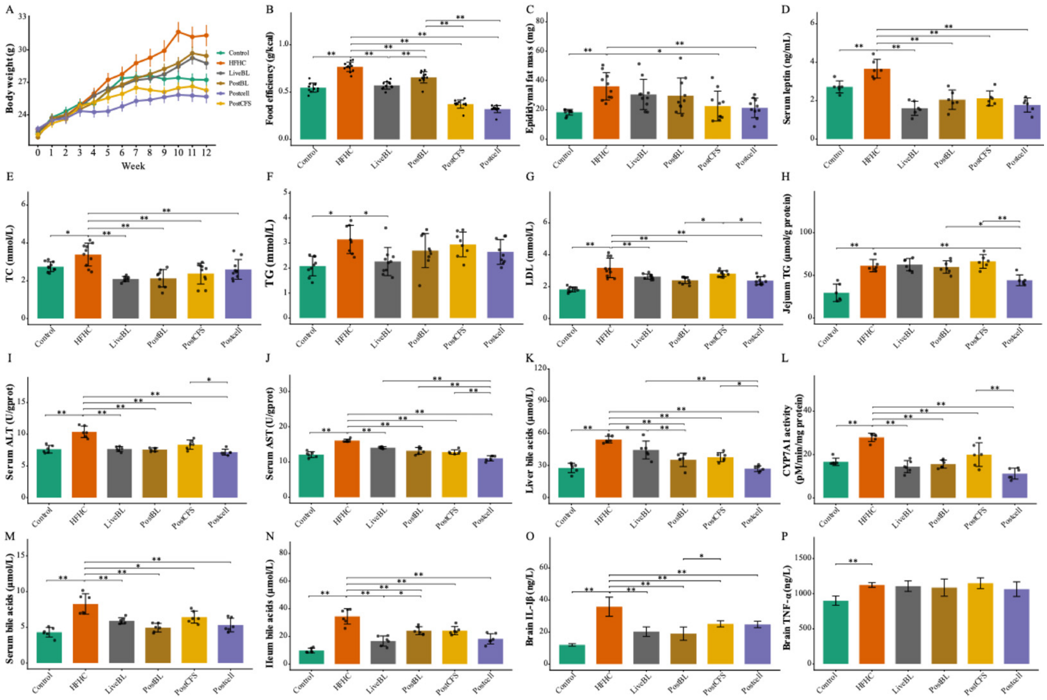

Figure S2. Effect of different FB 3-14 derived postbiotics in mice fed a high-fat and high-cholesterol diet. (**A**) Body weight over the entire experimental period, (**B**) Food efficiency, (**C**) Epididymal fat mass, (**D**) Serum leptin levels, (**E**) Serum TC, (**F**) Serum TG, (**G**) Serum LDL, (**H**) Jejunum TG, $n = 10$. (**I**) Serum ALT, (**J**) Serum AST, (**K**) Liver bile acids, (**L**) CYP7A1 activity, (**M**) Serum bile acids, (**N**) Ileum bile acids, (**O**) Brain IL-1β, (**P**) Brain TNF-α, $n = 6$. Data are presented as mean ± SD. * $P < 0.05$, ** $P < 0.01$.

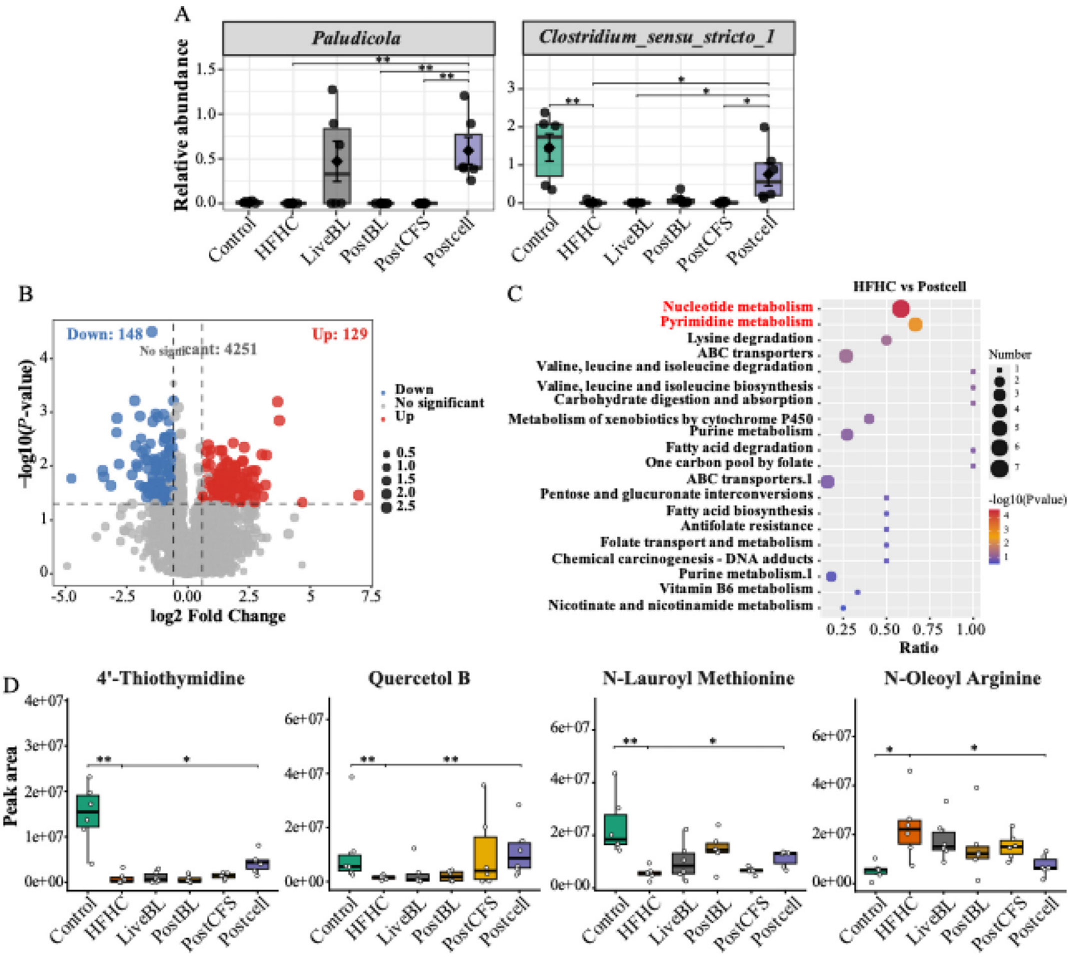

Figure S3. Effect of different FB 3-14 derived postbiotics on microbiome and metabolomic profiles. **(A)** Differentially abundant microbial taxa among groups (*n* = 6). **(B)** Volcano plot of differential metabolites. **(C)** KEGG pathway enrichment analysis. **(D)** The peak area of key differential metabolites (*n* = 6). Data are presented as mean ± SD. * $P < 0.05$, ** $P < 0.01$.

Table S1. Histological Score of Liver Tissue.

Score	Description
0	Normal liver architecture, no obvious steatosis or inflammatory infiltration
1	Mild hepatocyte swelling/vacuolar degeneration or slight inflammatory infiltration
2	Moderate vacuolar degeneration/steatosis with focal inflammatory infiltration
3	Marked hepatocyte injury, moderate inflammatory infiltration, and partial lobular architecture disruption
4	Severe diffuse hepatocyte injury, extensive inflammatory infiltration, and obvious lobular architecture disruption

Table S2. Top 50 annotated features detected in the DH extract across three independently prepared batches (negative-ion).

Name	Formula	RT (min)	Average peak area	Relative concentrations (μg/mL), Mean ± SD (μg/mL)
Citric acid	$C_6H_8O_7$	1.119	7659990060	1159.73 ± 8.18
Sucrose	$C_{12}H_{22}O_{11}$	0.783	5064533810	766.78 ± 10.90
DL-Malic acid	$C_4H_6O_5$	0.873	4144903804	627.54 ± 28.88
**Hesperidin**	$\mathbf{C_{28}H_{34}O_{15}}$	**7.171**	**2995628938**	**453.54 ± 4.88**
1,4-bischloromethyl-naphthalene	$C_{12}H_{10}Cl_2$	0.97	2357836086	356.98 ± 1.50
Isoquercitrin	$C_{21}H_{20}O_{12}$	6.71	2232645570	338.02 ± 11.11
Quercetin 3-O-β-D-Glucuronide	$C_{21}H_{18}O_{13}$	6.748	2115776950	320.33 ± 9.24
Gluconic acid	$C_6H_{12}O_7$	0.777	2111302912	319.65 ± 1.77
2-{4-[(6-Chloro-1,3-benzoxazol-2-yl)oxy]phenoxy}-N-(2-fluorophenyl)-N-methylpropanamide	$C_{23}H_{18}ClFN_2O_4$	0.887	1990851487	301.42 ± 1.82
4,7-Dinitro-2-oxo-5-phenyl-2,3,4,5-tetrahydro-1H-1,4-benzodiazepin-5-yl acetate	$C_{17}H_{14}N_4O_7$	5.171	1524103178	230.75 ± 4.65
**Narirutin**	$\mathbf{C_{27}H_{32}O_{14}}$	**6.924**	**1511797820**	**228.89 ± 2.01**
Quinic acid	$C_7H_{12}O_6$	0.816	892252119.5	135.09 ± 0.67
L-α-Glutamyl-L-histidyl-L-tryptophyl-L-seryl-L-tyrosylglycyl-L-leucyl-N~5~-isopropyl-L-ornithyl-L-prolylglycinamide	$C_{57}H_{81}N_{15}O_{14}$	9.356	711095461	107.66 ± 3.74
(−)-pinellic acid	$C_{18}H_{34}O_5$	9.931	708973060.3	107.34 ± 0.97
(+)-Catechin hydrate (+)	$C_{15}H_{14}O_6$	5.35	704631183.9	106.68 ± 0.62
(2R,4R)-4-Amino-1-(3,5-dinitrobenzyl)-2,4-pyrrolidinedicarboxylic acid	$C_{13}H_{14}N_4O_8$	1.085	704589846.1	106.68 ± 2.76
N-{[(2-Methyl-2-propanyl)oxy]carbonyl}-L-α-aspartyl-L-alanyl-L-α-aspartyl-L-aspartic acid	$C_{20}H_{30}N_4O_{13}$	0.813	680283825.5	103.00 ± 0.33
Epicatechin	$C_{15}H_{14}O_6$	5.892	655014957.8	99.17 ± 0.67
3,5-Di(2-pyrazinyl)-4H-1,2,4-triazol-4-amine	$C_{10}H_8N_8$	0.863	629603823.5	95.32 ± 0.99
3-Furoic acid	$C_5H_4O_3$	1.119	547497991.3	82.89 ± 1.64
4-Oxoproline	$C_5H_7NO_3$	1.203	545894157.2	82.65 ± 3.07
2-(4-Ethylphenyl)-2-oxoethyl 3-(4-methyl-1,3-dioxo-1,3-dihydro-2H-pyrrolo[3,4-c]quinolin-2-yl)benzoate	$C_{29}H_{22}N_2O_5$	0.781	530698322.4	80.35 ± 0.28
(±)-2-Hydroxyglutaric acid	$C_5H_8O_5$	0.868	511116984.3	77.38 ± 2.80
1-Salicylate glucuronide	$C_{13}H_{14}O_9$	4.925	471545698	71.39 ± 0.64
Orsellinic acid	$C_8H_8O_4$	5.636	435979670	66.01 ± 1.02
(2E,6E)-8-Anilino-3,7-dimethyl-2,6-octadien-1-yl trihydrogen diphosphate	$C_{16}H_{25}NO_7P_2$	0.804	431618523.3	65.35 ± 1.20

Quercetin 3-O-sambubioside	$C_{26}H_{28}O_{16}$	6.259	428154409.7	64.82 ± 0.75
Astragalin	$C_{21}H_{20}O_{11}$	7.1	420104901.1	63.60 ± 2.54
2-C-methylerythritol 4-phosphate	$C_5H_{13}O_7P$	0.764	394920425.9	59.79 ± 1.57
5-[(2-{[(3S)-5-{[(2S)-2-Amino-2-carboxyethyl]amino}-3-carboxy-3-hydroxy-5-oxopentanoyl]amino}ethyl)amino]-2,5-dioxopentanoic acid	$C_{16}H_{24}N_4O_{11}$	0.823	381463534.9	57.75 ± 4.59
Maleic acid	$C_4H_4O_4$	0.873	376150820.5	56.95 ± 3.60
Narcissoside	$C_{28}H_{32}O_{16}$	6.917	348665833	52.79 ± 0.43
Undecyl β-D-glucopyranoside	$C_{17}H_{34}O_6$	17.387	341320457.7	51.68 ± 1.69
4-Chloro-3-[(cyclopropyl{[(3R,4S)-4-{4-[2-(2,6-dichloro-4-methylphenoxy)ethoxy]phenyl}-3-piperidinyl]carbonyl}amino)methyl]benzyl methylcarbamate	$C_{34}H_{38}Cl_3N_3O_5$	7.168	326478241.3	49.43 ± 1.75
N-{1-[3-(Dimethylamino)propyl]-1H-benzimidazol-2-yl}cyclohexanecarboxamide	$C_{19}H_{28}N_4O$	9.582	305967168.6	46.32 ± 0.77
Azelaic acid	$C_9H_{16}O_4$	7.521	269883519.5	40.86 ± 0.54
Dehydroascorbic acid	$C_6H_6O_6$	1.173	263855530.7	39.95 ± 0.20
**Procyanidin B2**	$\mathbf{C_{30}H_{26}O_{12}}$	**5.172**	**234821875.4**	**35.55 ± 0.53**
Higenamine	$C_{16}H_{17}NO_3$	4.801	209206029.7	31.67 ± 1.66
Poncirin	$C_{28}H_{34}O_{14}$	8.453	184814294.1	27.98 ± 0.97
D-Saccharic acid	$C_6H_{10}O_8$	0.906	169232906.2	25.62 ± 0.63
Raffinose	$C_{18}H_{32}O_{16}$	0.825	157269373.4	23.81 ± 2.18
L-Tryptophan	$C_{11}H_{12}N_2O_2$	4.562	157037124.1	23.78 ± 0.37
Ferulic acid	$C_{10}H_{10}O_4$	6.958	148910870.8	22.55 ± 0.38
Vicenin II	$C_{27}H_{30}O_{15}$	5.7	144669877.9	21.90 ± 0.58
Rutin	$C_{27}H_{30}O_{16}$	6.5	138666021.8	20.99 ± 2.67
**Ginsenoside Rf**	$\mathbf{C_{42}H_{72}O_{14}}$	**9.292**	**137684574.8**	**20.85 ± 0.51**
Methylmalonic acid	$C_4H_6O_4$	1.347	133593716.5	20.23 ± 0.73
Citraconic acid	$C_5H_6O_4$	1.17	117086238.5	17.73 ± 0.73
Kaempferol-3-O-rutinoside	$C_{27}H_{30}O_{15}$	6.852	110632897.5	16.75 ± 0.67

Table S3. Top 50 annotated features detected in the DH extract across three independently prepared batches (positive-ion).

Name	Formula	RT (min)	Average peak area	Relative concentrations (μg/mL), Mean ± SD (μg/mL)
**Nuciferine**	**$C_{19}H_{21}NO_2$**	**7.924**	**2.8086E+10**	**276.51 ± 4.37**
DL-Arginine	$C_6H_{14}N_4O_2$	0.81	2.1354E+10	210.23 ± 2.66
Cinnyl Cinnamate	$C_{18}H_{16}O_2$	7.774	9152008207	90.10 ± 3.03
Betaine	$C_5H_{11}NO_2$	0.867	9064962518	89.24 ± 0.37
N-(4-Hydroxy-3-nitrophenyl)-2-[(1-methyl-2,4,6-trioxohexahydro-5-pyrimidinyl)carbonyl]hydrazinecarboxamide	$C_{13}H_{12}N_6O_8$	0.787	7629741530	75.11 ± 4.01
Choline	$C_5H_{13}NO$	0.765	7043209066	69.34 ± 4.96
Stachydrine	$C_7H_{13}NO_2$	0.916	5639166581	55.52 ± 3.13
(S)-(−)-4-Isopropyl-5,5-diphenyl-2-oxazolidinone	$C_{18}H_{19}NO_2$	6.865	3702249983	36.45 ± 0.80
10-Propylacridinium	$C_{16}H_{16}N$	5.635	3472737975	34.19 ± 0.65
Piperine	$C_{17}H_{19}NO_3$	6.577	2870590017	28.26 ± 0.82
Reboxetine	$C_{19}H_{23}NO_3$	6.292	2456044236	24.18 ± 0.28
Hesperidin	$C_{28}H_{34}O_{15}$	7.176	2163422741	21.30 ± 0.54
Trigonelline HCl	$C_7H_7NO_2$	0.938	2156964910	21.24 ± 0.35
2-Pyrrolidinecarboxylic acid	$C_5H_9NO_2$	0.872	2143621572	21.10 ± 0.67
Adenosine	$C_{10}H_{13}N_5O_4$	1.231	2050878883	20.19 ± 0.11
L-Leucine	$C_6H_{13}NO_2$	1.352	1830499499	18.02 ± 0.61
Isoquercitrin	$C_{21}H_{20}O_{12}$	6.725	1702733721	16.76 ± 0.99
L-Pyroglutamic acid	$C_5H_7NO_3$	1.145	1664683783	16.39 ± 0.86
Nobiletin	$C_{21}H_{22}O_8$	11.175	1454655188	14.32 ± 0.65
Ligustilide	$C_{12}H_{14}O_2$	12.51	1252522266	12.33 ± 0.30
6-O-[N~5~-(Diaminomethylene)-D-ornithyl]-α-D-galactopyranose	$C_{12}H_{24}N_4O_7$	0.823	1244671680	12.25 ± 0.31
Apomorphine	$C_{17}H_{17}NO_2$	6.713	1233629756	12.14 ± 0.69
trans-3-Indoleacrylic acid	$C_{11}H_9NO_2$	4.557	1213386573	11.95 ± 0.25
Quercetin 3-O-β-D-Glucuronide	$C_{21}H_{18}O_{13}$	6.757	1178977796	11.61 ± 0.58
Morin	$C_{15}H_{10}O_7$	6.73	979525633	9.64 ± 0.46
(1S,3aS,3bS,9aR,9bS,11aS)-N-(Dicyclohexylmethyl)-9a,11a-dimethyl-7-oxo-2,3,3a,3b,4,5,7,8,9,9a,9b,10,11,11a-tetradecahydro-1H-cyclopenta[i]phenanthridine-1-carboxamide	$C_{32}H_{50}N_2O_2$	9.368	974913844	9.60 ± 0.35
Cinnamic acid	$C_9H_8O_2$	2.228	956505523	9.42 ± 0.32
2-(1,3-Dimethyl-2,6-dioxo-1,2,3,6-tetrahydro-purin-7-yl)-N,N-diethyl-acetamide	$C_{13}H_{19}N_5O_3$	1.315	897737614	8.84 ± 0.27
Narirutin	$C_{27}H_{32}O_{14}$	6.927	858459792	8.45 ± 0.53
Glochidone	$C_{30}H_{46}O$	7.452	832635592	8.20 ± 0.55

Phenylacetylene	C_8H_6	2.231	825613115	8.13 ± 0.89
Cyclo(D-leucyl-L-leucyl-L-leucyl-L-leucyl-L-leucyl-L-leucyl)	$C_{36}H_{66}N_6O_6$	7.193	784484239	7.72 ± 0.26
Iminodimethanethiol	$C_2H_7NS_2$	0.674	780406949	7.68 ± 0.81
Pogostone	$C_{12}H_{16}O_4$	7.903	777808221	7.66 ± 0.31
ligustroflavone	$C_{33}H_{40}O_{18}$	9.347	742678981	7.31 ± 0.24
N-(2-AMINOETHYL)ETHYLENEUREA	$C_5H_{11}N_3O$	0.943	733974633	7.23 ± 1.14
Bis(2,2-dihydroxyethyl) hydrogen phosphate	$C_4H_{11}O_8P$	0.771	721539598	7.10 ± 0.21
(2-{(3R,5R)-3-Amino-6-[(2-aminoethyl)amino]-5-hydroxyhexanoyl}-1-methylhydrazino)acetic acid	$C_{11}H_{25}N_5O_4$	0.799	706534451	6.96 ± 0.32
Hesperetin	$C_{16}H_{14}O_6$	7.175	604580622	5.95 ± 0.04
Isosakuranin	$C_{22}H_{24}O_{10}$	7.177	589725931	5.81 ± 0.10
5-Hydroxymethylfurfural	$C_6H_6O_3$	0.95	563758817	5.55 ± 0.13
Tangeretin	$C_{20}H_{20}O_7$	11.697	562897719	5.54 ± 0.31
D-(+)-Pipecolinic acid	$C_6H_{11}NO_2$	1.032	523849899	5.16 ± 0.13
(+)-Catechin hydrate (+)	$C_{15}H_{14}O_6$	5.902	465231982	4.58 ± 0.17
p-Coumaric acid	$C_9H_8O_3$	1.236	436179247	4.29 ± 0.29
Cianidanol	$C_{15}H_{14}O_6$	5.351	430569372	4.24 ± 0.49
Cynaroside	$C_{21}H_{20}O_{11}$	7.122	421359394	4.15 ± 0.46
Synephrine	$C_9H_{13}NO_2$	1.089	420664698	4.14 ± 0.07
**Hordenine**	**$C_{10}H_{15}NO$**	**1.604**	**394318078**	**3.88 ± 0.08**
L-Glutamic acid	$C_5H_9NO_4$	0.887	359386572	3.54 ± 0.28
